# Exploring *α*-Lipoic Acid Based Thermoplastic Silicone Adhesive: Towards Sustainable and Green Recycling

**DOI:** 10.3390/polym16233254

**Published:** 2024-11-22

**Authors:** Jiaqi Wang, Zhaoyutian Chu, Sijia Zheng

**Affiliations:** Key Laboratory of Advanced Textile Materials and Manufacturing Technology and Engineering Research Center for Eco-Dyeing & Finishing of Textiles, Ministry of Education, Zhejiang Sci-Tech University, Hangzhou 310018, China; 2020327130021@mail.zstu.edu.cn (J.W.); 2023327130031@mails.zstu.edu.cn (Z.C.)

**Keywords:** *α*-lipoic acid, ring opening polymerization, silicone adhesives, H-bonding, disulfide, recyclable thermosets

## Abstract

Considering the demand for the construction of a sustainable future, it is essential to endow the conventional thermoset silicone adhesive with reuse capability and recyclability. Although various research attempts have been made by incorporating reversible linkages, developing sustainable silicone adhesives by natural linkers is still challenging, as the interface between the natural linker and the silicone is historically difficult. We exploited the possibility of utilizing *α*-lipoic acid, a natural linker, to construct a sustainable silicone adhesive. Via the simultaneous ring-opening reaction between the COOH and epoxide-functionalized silicone and the polymerization of the *α*-lipoic acid, the resulting network exhibited dynamic properties. The shear strength of the LASA90 presented strong adhesion (up to 88 kPa) on various substrates including steel, aluminum, PET, and PTFE. Meanwhile, reversible adhesion was shown multiple times under mild heating conditions (80 °C). The rheology, TG-DTA, DSC, and ^1^H NMR showed that the degradation of the LASA occurred at 150 °C via the retro-ROP of the five-membered disulfide ring, indicating their recyclability after usage. Conclusively, we envision that a silicone adhesive based on *α*-lipoic acid as a natural linker is more sustainable than conventional silicone thermosets because of its desired properties, strong adhesion, reversibility, and on-demand heat degradation.

## 1. Introduction

Silicone adhesives, presenting many exceptional properties, are widely used in various fields including household sealants, automobiles, electronics, medical devices, and aerospace [1,2,3]. Conventional silicone networks are thermosets and, therefore, are not able to be reused, recycled, or easily degradable in the environment. Parts joined by silicone adhesives are typically discarded without being dismantled. In this context, silicones are not readily “green”, and there is an urgent demand for the development of sustainable silicone adhesives to echo the commitment to building a greener future.

The primary requirements for sustainable silicone adhesives are (1) reversible adhesion that can provide sufficient adhesive strength under multiple uses; (2) removable at a desired time; and (3) degradable in the environment at the end of life [4]. In the state of the art, incorporation of a reversible linkage offers potential solutions. Supramolecular (ionic, hydrogen bonding [5,6], aromatic stacking [7,8], interlocking [9,10]) and dynamic covalent chemistry (C=N [11], S-S [12], Se-Se [13], etc.) offer a tremendous toolbox for the designing of smart materials that are stimuli-responsive, recyclable, re-processable, and adaptable.

Furthermore, with the ongoing environmental concerns, whether synthetic silicone polymers are environmentally friendly materials has caused considerable controversy. Although the products of silicone decomposition are sand, carbon dioxide, and water [14], the EU has set strict limits on siloxane cyclics (D_4_, D_5_, and D_6_) due to their potential threats to marine life [15,16]. One way to reduce the environmental impacts of materials is to dilute them with natural components [17,18,19]. For example, silicone foams with lignin as a reinforcing agent and the boiling agent were achieved by the Piers–Rubinsztajn reaction [20]. Another phenolic compound, eugenol, was also reported to synthesize silicone oil, foam, or elastomer by sequence control [21,22]. Other emblematic green compounds (e.g., vanillin [23], vitamin E [21,24], saccharide [17], coumarin [25], and tannic acid [26]) were also successfully induced into the silicone for various research purposes. We reasoned that incorporating natural products with dynamic linkages could simultaneously dilute the impact of silicones on the environment while leading to materials with reversible properties.

The ingredient *α*-lipoic acid, a natural biomolecule that is essential for aerobic metabolism, has attracted significant interest recently [27]. Especially due to the development of synthetic technology, it is now available as a prevalent dietary supplement and pharmaceutical drug. Many pioneering efforts represent the milestones of utilizing this cyclic disulfide with nature-derived dynamic properties [28]. The disulfide-mediated ring-opening polymerization (ROP) of thioctic acid was first reported by Zhang et al. in 2018 [29]. Since then, the same group has done a systematic study on developing dynamic polymer materials based on *α*-lipoic acid [30,31,32]. Thereafter, this disulfide-based dynamic chemistry enabled dynamic polymers with distinctive functional applications [33,34,35]. The incorporation of *α*-lipoic acid within silicone was first reported by Choi et al. in 2021 [36]. The *α*-lipoic acid was functionalized to silanol-terminated silicone via an esterification reaction, and the resulting elastomer allowed for light-mediated self-healing and on-demand chemical degradation. Furthermore, Noman et al. reported on silicone-lipoamide elastomers developed based on *α*-lipoic acid [37]. Notably, the degradation of these lipoic silicone elastomers undergoes a redox pathway. While not yet demonstrated, the synthesis and degradation process can be achieved via a simpler procedure that involves less chemical treatment.

**Figure 1:**
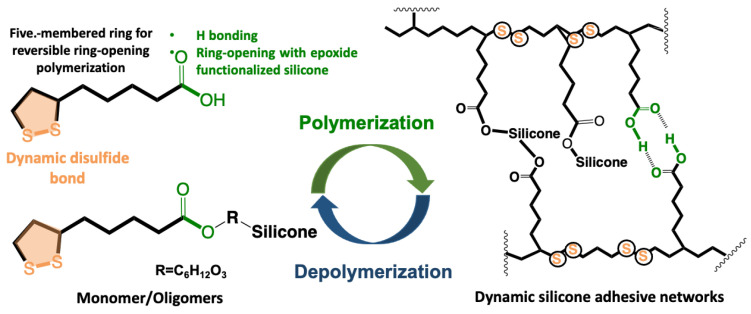
The schematic diagram of the design and reversibility of LASAs.

Inspired by the many milestones attained by those pioneer efforts, we proposed to expand the chemical toolbox for fabricating dynamic silicone materials, combining the features of dynamic covalent and noncovalent materials. The designed silicone adhesives can provide reversible adhesion during application and also easily degrade at the end of life. In this work, we present a fundamental approach for developing eco-friendly silicone-based adhesives. We introduced a simple synthetic strategy to form bottlebrush silicone adhesives via the two ring-opening reactions that occurred concomitantly. (1) The ring-opening reaction between epoxide functional silicone and COOH from *α*-lipoic acid. (2) The ring-opening polymerization of *α*-lipoic acid. To modulate the adhesion properties, the ratio of COOH to epoxide functionality was considered. The resulting network has two dynamic linkages—hydrogen bonding between COOH and disulfide bonds—and endows the designed network with excellent properties, such as good adhesion, reversibility, and on-demand heat degradation (Figure 1).

## 2. Materials and Methods

### 2.1. Materials

The materials *α*-lipoic acid (99%) and octamethylcyclotetrasiloxane (D_4_, >98.0%) were received from Leyan. Magnesium oxide, activated charcoal (AR. >200 mesh), allyl glycidyl ether (99%), 1,1,3,3-tetramethyldisiloxane (M^H^M^H^, 98%), and n-butyllithium solution (1.6 M in hexane) were purchased from Shanghai Aladdin Bio-Chem Technology Co., Ltd. (Shanghai, China). Karstedt’s catalyst solution (2% in xylene), trifluoromethanesulfonic acid (98%), and dimethylchlorosilane (95%) were received from Macklin Inc. (Shanghai, China). Isopropanol and n-hexane were obtained from Shanghai Lingfeng Chemical Reagent Co., Ltd. (Shanghai, China). Hexamethylcyclotrisiloxane (D_3_, >98.0%) and dry tetrahydrofuran (THF) were received from Energy Chemical. All chemicals were used as received without further purification.

### 2.2. Synthesis of Silicone Oils and Fabrication of LASAs

#### 2.2.1. Synthesis of *α*-Monoepoxypropoxypropyl-*ω*-Monobutyl Terminated Polydimethylsiloxane (Mono-PDMS-E)

**Scheme 1:**
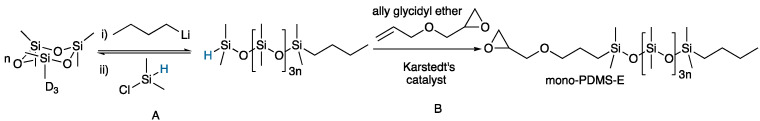
Schematic diagram of the synthesis of bis-PDMS-E via the D_4_ equilibration (Step A) and the hydrosilylation reaction (Step B).

Scheme 1, Step A: In a pre-dried 500 mL Schlenk flask, D_3_ (50.0 g, 0.23 mol) was added. The flask was connected with N_2_ and a vacuum line. The flask was pulled by vacuum and flushed with N_2_ three times. Dry THF (140 mL) solvent was injected into the flask through a long needle to dissolve the D_3_. The initiator, n-Butyllithium solution (13.1 mL, 1.6 M, 20.96 mmol), was added dropwise via a syringe. The capping agent, dimethylchlorosilane (0.47 mL, 41.92 mmol), was added dropwise 90 min after the initiation of the reaction. The reaction mixture was allowed to stir overnight, and the crude product was concentrated by rotary evaporation. The mixture was an opaque liquid with a white perception. The white lithium chloride salt was removed by filtration. Any impurities or excess cycles were distilled off at 160 °C for 120 min under reduced pressure, yielding transparent *α*-monohydride-*ω*-monobutyl terminated polydimethylsiloxane (Yield: 84%). The chemical structure and molecular weight were determined by ^1^H NMR: Mn = 1600 g mol^−1^ (Figure S1).

Scheme 1, Step B: *α*-monohydride-*ω*-monobutyl terminated polydimethylsiloxane (42.00 g, 2.6 × 10^−2^ mmol Si-H), allyl glycidyl ether (4.59 g, 4.0 × 10^−2^ mmol), and Karstedt’s solution (47.3 μL, 20 ppm Pt to reaction system) were mixed into a 250 mL dry round bottom flask. The mixture was stirred at 80 °C, under N_2_ protection, for 10 h. Subsequently, activated charcoal (10 g) was added and stirred overnight at room temperature to adsorb the Karstedt’s catalyst. The reaction mixture was filtered through a glass funnel filled with a layer of celite. The collected crude product was distilled at 160 °C for 120 min under reduced pressure. The final product mono-PDMS-E was a clear liquid. Yield 79%, Mn = 1750 g mol^−1^ (Figure S2).

#### 2.2.2. Synthesis of *α*, *ω*-Epoxypropoxypropyl Terminated Polydimethylsiloxane (Bis-PDMS-E)

**Scheme 2:**
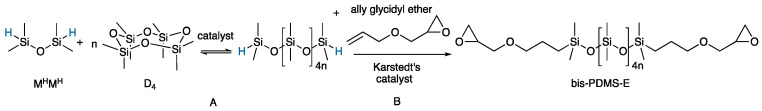
Schematic diagram of the synthesis of mono-PDMS-E via the D_3_ equilibration and (Step A) and the hydrosilylation reaction (Step B).

Scheme 2, Step A: The *α*, *ω*-hydride terminated polydimethylsiloxane was synthesized via an equilibration reaction. D_4_ (100.00 g, 0.34 mol), M^H^M^H^ (3.32 g, 2.36 × 10^−2^ mol), and trifluoromethanesulfonic acid (303.5 μL) were mixed in a 250 mL round bottom flask. The reaction mixture was stirred at room temperature for 10 h, followed by heating at 80 °C for 10 h. Subsequently, magnesium oxide (~ 5 g) was added to quench the catalyst. The mixture was filtered through a funnel layered with celite. The celite layer was rinsed with hexane to collect the adhered product. The hexane was removed by rotary evaporation, and the residual cyclics were removed by reduced pressure distillation at 160 °C for 120 min. The yield was 98%. The chemical structure was confirmed by ^1^H NMR, and the molecular weight: Mn = 4400 g mol^−1^ (Figure S3).

Scheme 2, Step B: The hydrosilylation reaction of *α*-monohydride-*ω*-monobutyl terminated polydimethylsiloxane (100 g, 2.28 × 10^−2^ mol hydride groups) with allyl glycidyl ether (3.98 g, 3.49 × 10^−2^ mol) and Karstedt’s solution (110 μL, 20 ppm Pt) was performed within a 250 mL flask. The reaction flask was connected to an N_2_ balloon via a syringe. The flask was heated in an 80 °C oil bath for 10 h. Once complete, 10 g activated charcoal was added and stirred overnight to remove the catalyst. The charcoal was removed by going through a funnel layered with celite. The celite layer was rinsed with hexane to collect the adhered product. The hexane was removed by rotary evaporation and the residual cyclics were removed by reduced pressure distillation at 160 °C for 120 min. The product bis-PDMS-E was a clear liquid. Yield 91.6%, Mn = 4100 g mol^−1^ (Figure S4).

#### 2.2.3. Preparation of LASAs

For Table S1, entry 1, the post-curing procedures were slightly different from the formulations with excess COOH (entries 2-4). First, *α*-lipoic acid (0.1949 g, 9.46 × 10^−4^ mol) and mono-PDMS-E (1.00 g, 5.68 × 10^−4^ mol of epoxide) were stirred at 120 °C for 4 h. Then, bis-PDMS-E (1.55 g, 3.79 × 10^−4^ mol of epoxide) was added into the reaction mixture. The reaction mixture was poured into the mold (80 mm × 40 mm × 2 mm or 80 mm × 40 mm × 0.5 mm) lined with a Teflon sheet. The post-curing procedure was heating at 150 °C for 2 h and 180 °C for 2 h. The cured product was a transparent brown elastomer.

**Scheme 3:**
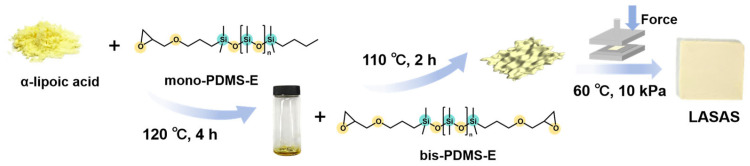
The schematic diagram for the fabrication process of LASAs.

As Table S1, entry 2-4, Scheme 3: a certain amount of *α*-lipoic acid was heated to 120 °C in a 30 mL glass vial. When the yellow solid turned into liquid, mono-PDMS-E (1.00 g, 5.68 × 10^−4^ mol of epoxide) was added and the mixture was allowed to be stirred at 120 °C for 4 h (Figure S5, path A). After 4 h, the reaction mixture turned into a homogenous opaque yellow liquid. Subsequently, bis-PDMS-E (1.55 g, 3.79 × 10^−4^ mol) was introduced into the reaction mixture and well-mixed by stirring with a spatula. After stirring, the mixture was heated at 110 °C for 2 h (Figure S5, path B). The resultant product was a sticky glue. Finally, the LASAs were molded by hotpress with a square stainless-steel mold (80 mm × 40 mm × 2 mm or 80 mm × 40 mm × 0.5 mm) at 60 °C, 10 kPa, for 5 min. The final products were opaque, yellowish sheets that could be cut into the required shape for characterization.

### 2.3. Characterization Methods

#### 2.3.1. Solvent Resistance

The solvent swelling ratio of the LASA90 was evaluated by immersing the LASA90 (0.2 g) into a glass vial containing 10 mL solvent. Several solvents with different polarities were examined (dichloromethane (DCM), toluene (PhMe), ethyl acetate (EAC), tetrahydrofuran (THF), ethanol (EtOH), methanol (MeOH), and DI water (H_2_O)). The samples were stored at room temperature for 14 days. The swelling ratios were calculated by the following equation:

(1) $$R=\frac{m_{0}-m_{i}}{m_{i}}\times 100\%.$$

where m_0_ is the weight after immersing in solvent for 14 days. The samples were wiped with Kimwipe before weighing. The variable m_i_ represents the initial sample weights.

#### 2.3.2. Shear Strength Tests

The LASA samples were cut into a square shape (width: 10 mm, length: 25 mm, thickness 0.5 mm). The sample was sandwiched in between two pieces of test substrates (steel, alumina, PET, PTFE, glass). The overlap area was 10 mm × 20 mm. The test sample joints were clamped with clips for 24 h before tests. The measurements were performed on a tensile tester (Guangdong Kejian Instrument Co., Ltd., Dongguan, China) and triplets with a strain rate of 10 mm·min^−1^. The shear strength was calculated using the following equation. The measurements were all conducted in triplicates. The average values and standard deviation were reported.

(2) $$Shear\;strength=\frac{F}{S}$$

where the F is the maximum force before the joint breaks. S is the overlap area of the two test substrates.

#### 2.3.3. Reversible Surface Adhesion Tests

The torn test joints with LASA (10 mm × 20 mm × 0.5 mm) were clamped with clips and heated at 80 °C for 1 h. The sample joints were annealed at room temperature for 24 h before tests. Subsequently, the shear strength of the recovered joints was tested by a tensile tester (Guangdong Kejian Instrument Co., Ltd., China) at a peeling speed of 10 mm·min^−1^. The shear strengths were calculated from Equation (2). Each test was repeated three times, and the average values were reported. Four cyclics were repeated in total.

#### 2.3.4. Thermoplastic Performance

A 1 cm length cut was made on LASA0 and LASA90 (thickness 2 mm). The sample was stored at room temperature for 1.5 h or heated to 100 °C for 1.5 h in an oven. The cuts on the samples before and after healing were observed under an optical microscope (EX20, Shunyu, Ningbo, China).

#### 2.3.5. Evaluation of Adhesion of LASA Under Different Humidities

To evaluate the influence of humidity on the adhesion properties of LASA, a piece of a LASA sample strip (width: 10 mm, length: 25 mm, thickness 0.5 mm) was sandwiched between two steel plates. The joints were clamped with two paper clips and placed in a constant temperature and humidity oven (STIK Co. Ltd., Shanghai, China) for 24 h before shear strength tests. The temperature was set at 25 °C, with specific humidity ranging from 20% to 98%. The shear test was performed on a tensile tester, and the strain rate was set as 10 mm·min^−1^. The shear strengths were calculated using Equation (2). All measurements were conducted in triplets.

#### 2.3.6. Rheology Tests

The LASA samples were cut into a round shape (diameter: 20 mm, thickness 2 mm) by a punch. The rheology behaviors of the LASA were performed in a rheometer (Mars40, Haake, Germany) equipped with a 20 mm flat plate. The constant force was set at 2 N to ensure contact of the sample with the plate. For the frequency swap, the tests were done at room temperature, and the strain was 1%. The frequency 0.1–100 Hz was examined. For the temperature swap, the loss modulus, storage modulus, viscosity, and tan *δ* were recorded under room temperature to 800 °C. The temperature ramp was set as 5 °C min^−1^. The swap frequency was 2 rad s^−1^, and the strain was 1%. The gelling temperatures of the LASAs were defined as the temperature at which the storage modulus (G′) intersected the loss modulus (G″) [38]. The G′ and G″ were recorded from temperature swap tests.

#### 2.3.7. Other Characterizations

Proton nuclear magnetic resonance (^1^H NMR): ^1^H NMR spectra were recorded on a nuclear magnetic resonance spectrometer (Bruker Avance 400 MHz, Bruker, Billerica, MA, USA). The spectra were measured by dissolving samples in deuterated solvents chloroform-d (proton impurity for solvent was calibrated to 7.26 ppm). Fourier-transform infrared spectroscopy (FTIR): Measurements were performed on a FTIR spectrometer (Nicolet iS20, Thermo Fisher Scientific, Waltham, MA, USA) equipped with an attenuated total reflectance (ATR) attachment. Thermogravimetric analysis (TG), differential thermal analysis (DTA), and derivative thermogravimetric (DTG) were recorded on a simultaneous TG-DSC apparatus (PerkinElmer STA 8000, Waltham, MA, USA) under N_2_ atmosphere. The rate for the temperature ramp was 10 °C min^−1^. Differential scanning calorimetry (DSC) was performed on a Netzsch DSC214 (Netzsh, BY, Selb, Germany) under N_2_ atmosphere. The rate for the temperature ramp was 10 °C min^−1^.

## 3. Results and Discussion

### 3.1. Formation and Characterization of LASA

The crosslinked networks of LASA were prepared by mixing the *α*-lipoic acid with *α*-monoepoxypropoxypropyl-*ω*-monobutyl terminated polydimethylsiloxane (mono-PDMS-E) and *α*, *ω*-epoxypropoxypropyl terminated polydimethylsiloxane (bis-PDMS-E) at different molar ratios (Table S1, Scheme 3, and Figure S1). The initial attempts suggested that the optimized condition for the epoxy ratio from mono-PDMS-E to bis-PDMS-E was 50:50 (Table S1, Figure S6). Activated by the ring strain of the five-membered rings in the *α*-lipoic acid, the disulfide bond readily undergoes self-polymerization heat initiation. Concomitantly, the ring-opening reaction of epoxide and COOH was expected to occur at an elevated temperature. The prepared LASA networks were named LASA0, LASA50, LASA75, and LASA90, with the last number referring to the excess COOH ratio versus the COOH that participated in crosslinking.

As in most of the cases of making composite materials, the main synthetic challenge associated with creating silicone networks with natural products was overcoming the silicone/*α*-lipoic acid interfaces. In our design, *α*-lipoic acid was first reacted with mono-PDMS-E to increase the compatibility between the two components. Then, bis-PDMS-E was added and cured at 110 °C to achieve the dynamic networks for LASA50, LASA75, and LASA90. It should be noted that although the reaction between epoxy and COOH is supposed to occur at 110 °C, the LASA0 pre-cure mixture remained a liquid after heating at 110 °C/2 h or 150 °C/2 h (Figure S7). The curing occurred at “150 °C/2 h + 180 °C/2 h” (Figure S7). The results suggested that the COOH-rich environment may self-catalyze the ring-opening of epoxy, leading to the desired network under mild conditions.

**Figure 2:**
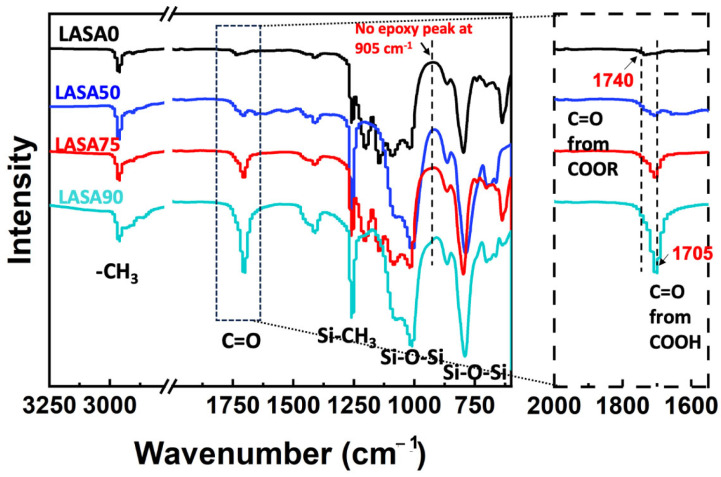
The FTIR spectra of LASA0, LASA50, LASA75, and LASA90.

The crosslinking reaction between the epoxy and COOH and the chemical structure was confirmed by Fourier-transform infrared (FTIR, Figure 2). The FTIR spectra displayed all the characteristic peaks for silicone. The peaks at 1250 cm^−1^ and 850 cm^−1^ were the bending vibration and rocking vibration of Si-CH_3_. The broad peaks at 1080 cm^−1^ and 1000 cm^−1^ were assigned to the Si-O-Si, and the peaks presented at 2950 cm^−1^ were stretching vibrations of CH_3_. The FTIR spectra of the LASA showed that there were no epoxy peaks at 905 cm^−1^. In LASA0, the carbonyl group (C=O) presented at 1740 cm^−1^, which is higher than the C=O from COOH in *α*-lipoic acid (1704 cm^−1^). Meanwhile, in LASA50, LASA75, and LASA90, an increased amount of residual COOH was designed within the network. Correspondingly, the peak intensities at 1705 cm^−1^ increase, consistent with the feedstock in the formulation. The increasing residual COOH will, on the one hand, form dimers within the bulk, endowing LASAs with robust and reversible coherence. On the other hand, the association/dissociation of COOH dimers will introduce free COOH on the substrate/LASAs interfaces, rendering the interfacial with adhesion properties.

**Figure 3:**
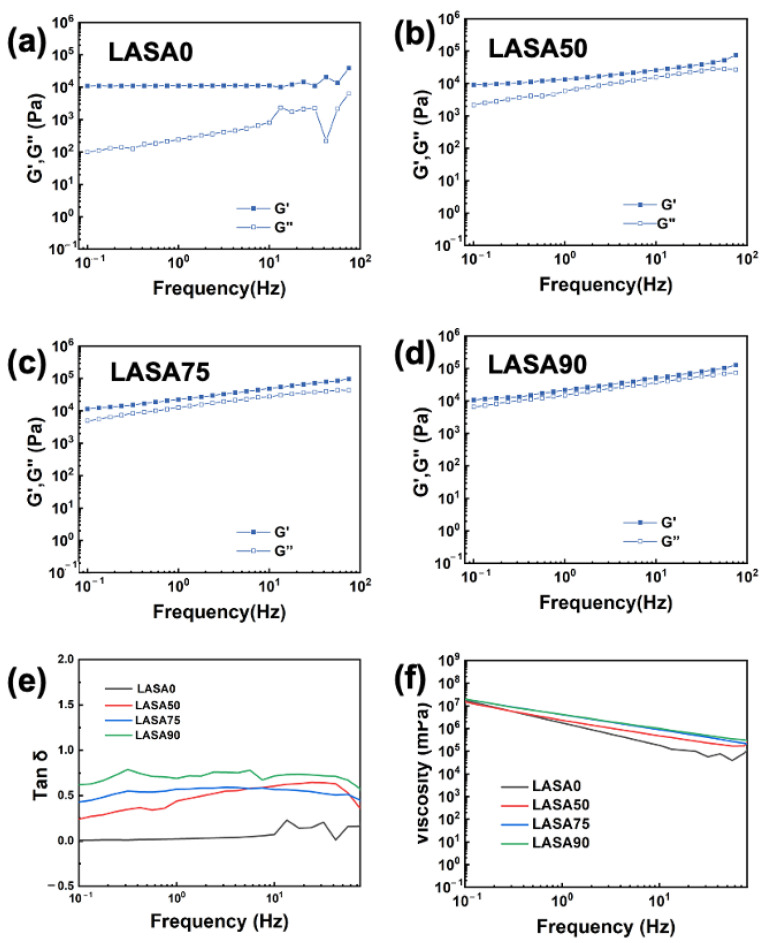
The storage modulus (G′) and loss modulus (G″) of the LASAs as a function of frequency. (**a**) LASA0, (**b**) LASA50, (**c**) LASA75, (**d**) LASA90. (**e**) The tan *δ* as a function of frequency. (**f**) Viscosity of the LASAs as a function of frequency.

The crosslinking of the LASAs was evaluated by rheological behavior Figure 3. The frequency sweeps were conducted to characterize the network. As shown in Figure 3a, the storage modulus (G′, 1.1 × 10^5^–1.3 × 10^4^ Pa) was all higher than the corresponding loss modulus (G″, 1.0 × 10^2^–6.3 × 10^3^ Pa), indicating the formation of crosslinked networks. The difference between the G′ and G″ dropped significantly relative to that of LASA0. Specifically, the G′ (1.0 × 10^4^–1.4 × 10^5^ Pa) and G″ (6.5 × 10^3^–1.6 × 10^4^ Pa) values were close within the entire frequency regime for LASA90 (Figure 3d). Correspondingly, the tan *δ* values, which are the ratios of G″/G′, were LASA0 < LASA50 < LASA75 < LASA90 (Figure 3f). Specifically, the tan *δ* of LASA90 was 0.6–0.7, indicating the improved damping performance of the LASA90. These results can be attributed to the increase of H-bonding concentration within the LASA50, LASA75, and LASA90. The impact energy can be dispersed via H-bonding shifting. Furthermore, the viscosity of the LASAs was close, and slight decreases were observed in the high frequency range. Considering the different curing behavior of LASA0 and LASAs with residual COOH, it can be concluded that both the rates of the reactions and the ultimate network can be well controlled by the stoichiometric ratios of the COOH to epoxy.

**Figure 4:**
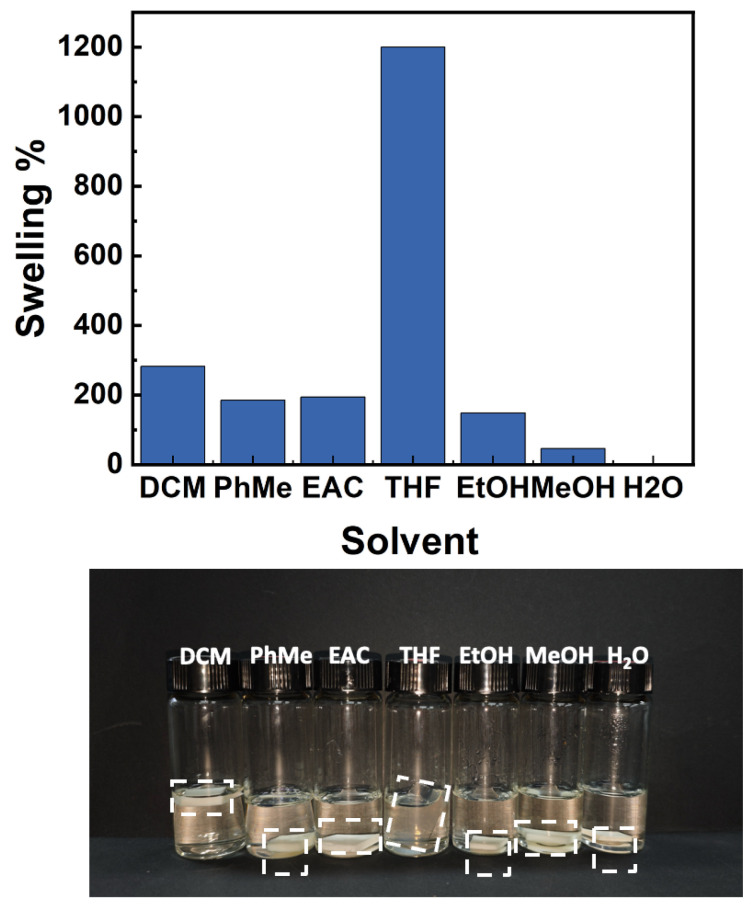
The solvent-resistant behavior of LASA90 against various solvents at 14 days.

During its service life, it is inevitable that a LASA may come into contact with some solvent during application. Herein, the swelling ratios of the LASA90 within different solvents were characterized to reveal its solvent-resistant properties. Solvents with different polarities were chosen. The result of the solvent-resistant test showed that LASA90 only swelled and remained intact after being immersed in the solvent for 14 days (Figure 4). Furthermore, the swelling ratio of LASA90 in DCM, PhMe, EAC, THF, EtOH, MeOH, and H_2_O were 282%, 185%, 194%, 1200%, 148%, 46%, and 0%. Herein, the dynamic network with both covalent linkages and noncovalent linkages demonstrated good chemical resistance to organic solvents with different polarities.

### 3.2. Surface Adhesion Properties

**Figure 5:**
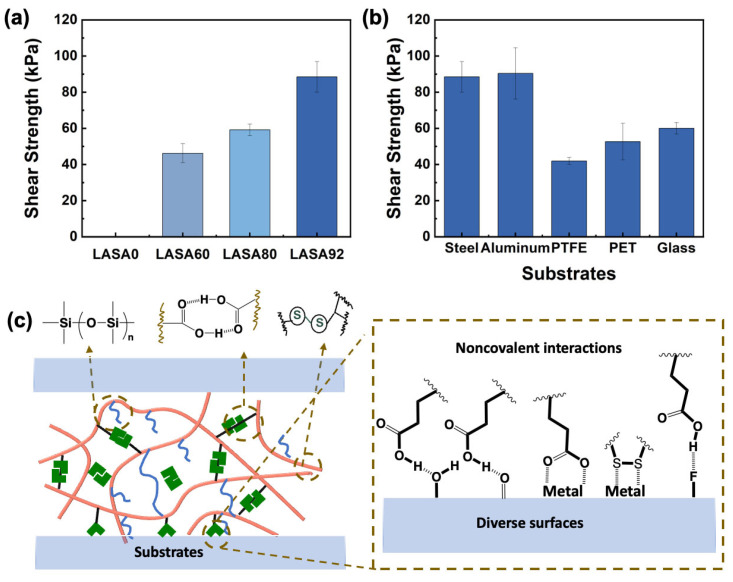
(**a**) Shear strength between the LASA and steel substrates. The shear test measurement was not applicable for LASA0. (**b**) Shear strength of LASA90 on diversified substrates. (**c**) Schematic representation of the LASA and the noncovalent interaction established between diversified substrates (H-bonding, metal coordination, and Van der Waals force).

Due to the low surface tension of silicone-based materials, adhering silicone-based materials to surfaces was historically difficult to achieve. Owing to the abundant COOH groups within the network, the LASA was expected to show high affinities to various substrates. The LASAs were sandwiched between two pieces of substrates and clamped with a paper clip at room temperature to achieve sufficient contact. Quantitively, the shear strength of the LASAs displayed a linear positive relationship with the residual COOH within the network (Figure 5a). The shear strength for LASA90 achieved 88 kPa, the highest among the formulations examined. This result can be ascribed to the high density of H-bondings provided by COOH. The complementary hydrogen bonding provided by the COOH dimers could provide sufficient cohesion strength, and the dissociated COOH at the LASA/substrates could provide interfacial adhesion.

The adhesion strength of LASA90 on various substrates was subsequently evaluated (Figure 5b). The LASA90 showed universal adhesion to various substrates. The LASA90 exhibited good shear strength on the metal surface and mediocre adhesion on the glass and PET substrates. Promisingly, the adhesion strength of LASA90 on PTFE substrates was 42 MPa. These results can be ascribed to the H-bondings with the hydroxyl groups of hydrophilic groups and the fluorine groups on PTFE. Moreover, the sulfur-rich LASA provided an additional affinity to metallic surfaces (Figure 5c).

**Figure 6:**
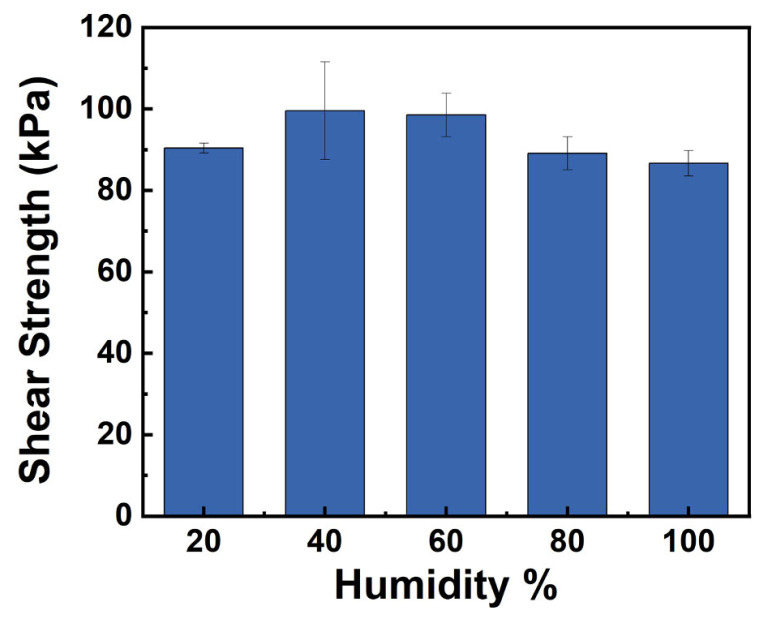
Shear strength between the LASA90 and steel substrates in different humidity.

The universal application of the LASA within environments of different humidities was also investigated, as the dimerization of the COOH groups was expected to be seriously related to the environmental humidity. When the number of dissociated COOH groups increases, the possibility of COOH participating in interfacial bonding will increase. Surprisingly, as shown in Figure 6, the shear strengths of the LASA90 did not show statistical changes under the 20%-100% humidity regime. This might be reasonably explained by the hydrophobicity of the silicone components that inhibited the penetration of water into the LASA, leading to the dissociation of the COOH dimer and subsequent deterioration in shear strength.

### 3.3. Reversible Adhesion Properties

**Figure 7:**
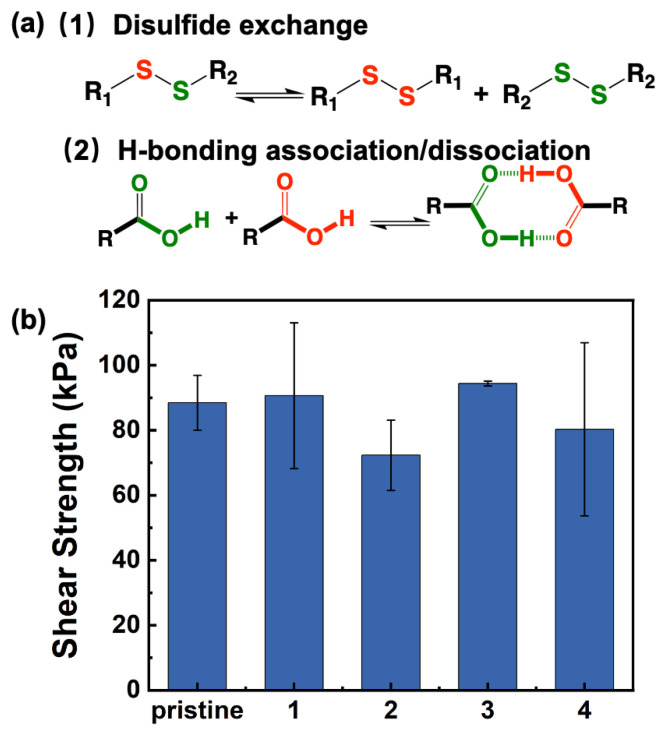
(**a**) Two dynamic mechanisms for the LASA90 under heat conditions. (**b**) Shear strength of LASA90 after multiple tests.

In the LASA networks, there are both dynamic bonding and noncovalent bonds (Figure 7a), giving the LASA excellent reversibility. The reusability of the adhesion of the LASA90 was examined by reassembling the separated joints at 80 °C. The shear strength of the LASA90 manifested no significant deterioration after 5 cycling experiments (Figure 7b), suggesting excellent reusability due to the dynamic nature of the LASA90 network.

**Figure 8:**
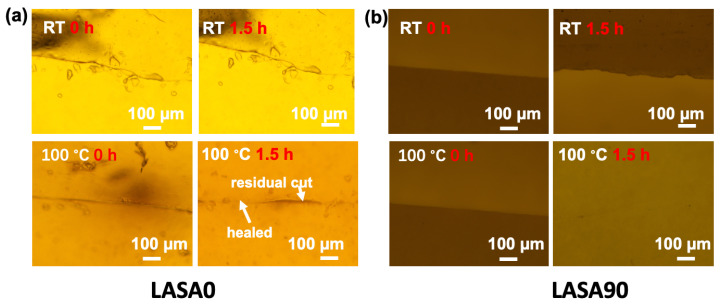
Microscope photos of the cuts on LASA0 (**a**) and LASA90 (**b**). The LASA0 and LASA90 were allowed to heal at room temperature or 100 °C for 90 min.

Formulations with and without residual COOH were designed to reveal the mechanism of the reversibility, and the mechanism was verified by the dynamic performance under heat conditions. As shown in Figure 8, a cut was made through the sample, and changes in the cut were observed after healing at room temperature or 100 °C. Both LASA0 and LASA90 did not present healing performance when kept at room temperature. Promisingly, when the samples were kept at an elevated temperature (100 °C, 90 min), the cut made on the LASA0 slightly healed due to the disulfide exchange. Furthermore, the cut made on the LASA90 was healed after heating at 100 °C for 90 min due to the presence of heat-labile H-bonds. Herein, the H-bonding was considered the major reversible mechanism.

### 3.4. Recycle and Thermal Degradation of LASA

#### 3.4.1. The Green Recycling via Retro-ROP of Disulfide Linkages

The material *α*-lipoic acid is known to participate in ROP reactions once its melting temperature is achieved (70 °C), while the depolymerization to monomers or oligomers is supposed to occur at 180 °C [28]. Based on this understanding, the recycling of the designed LASA was expected to be achieved via the reversible dynamic disulfide bond, where the crosslinked network can be depolymerized into five-membered disulfide rings. The recycled monomers and oligomers were expected to re-crosslink into elastomeric materials for new applications.

**Figure 9:**
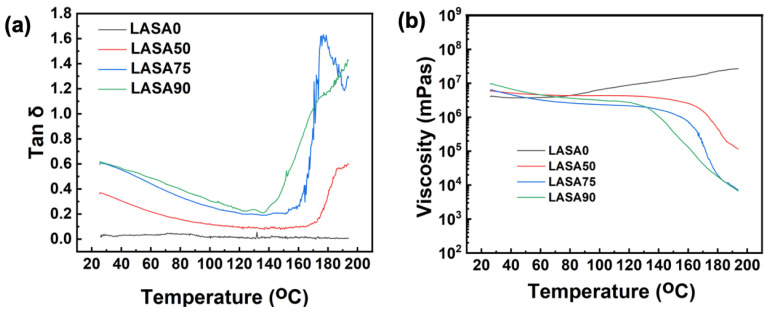
(**a**) Tan *δ* and (**b**) viscosity of the LASAs as a function of temperature.

To exploit the possibility of retro-ROP, the rheological behavior of the LASAs as a function of temperature was studied by a rheometer (Mars40, Haake, Germany). Liquidation of the LASA was expected to happen when the retro-ROP occurred. The tan *δ* of the LASA50, LASA75, and LASA90 displayed a sharp increase at the elevated temperature (Figure 9a). With the increase of the H-bonding concentration, the temperature for the initiation temperature for tan *δ* shifted to a lower temperature: 175 °C for LASA50, 160 °C for LASA75, and 150 °C for LASA90. Meanwhile, the viscosities of the LASA50, LASA75, and LASA90 followed the same trend, where a sudden decrease was observed at 170 °C, 160 °C, and 130 °C, respectively (Figure 9b). These results indicate that the retro-ROP of LASA50, LASA75, and LASA90 may occur during heating, facilitating the liquidation of the materials. Surprisingly, it is worthwhile to note that the LASA0 exhibited typical rheology for non-degradable thermosets under 200 °C. The tan *δ* and viscosity of the LASA0 were kept constant during the heating process (Figure 9). Due to the lack of dynamic mechanisms (no re-ROP of cyclic disulfide and no residual COOH for H-bonding), the LASA0 was a thermoset that was not able to be recycled.

**Figure 10:**
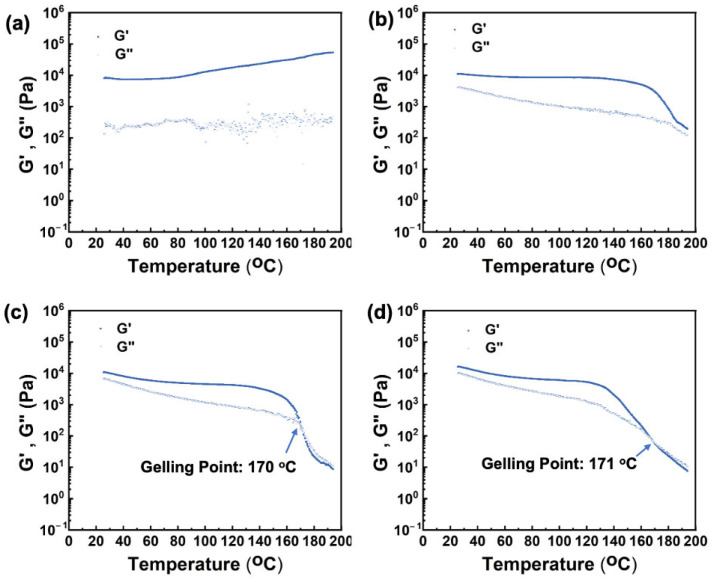
The gelling points of the LASAs determinate by G′ and G″. (**a**) LASA0, (**b**) LASA50, (**c**) LASA75, (**d**) LASA90.

Meanwhile, the storage modulus and the loss modulus of the LASAs were also recorded by a rheometer (Figure 10). The storage modulus of the LASA0 processed the storage modulus (G′) at the platform (8.0 × 10^3^–5.3 × 10^4^ Pa) under the temperature range (room temperature to 195 °C), several orders of magnitude higher than the loss modulus (G″, 1.3 × 10^2^–6.2 × 10^2^ Pa). However, with the increase of COOH originating from *α*-lipoic acid, the moduli of the LASA50, LASA75, and LASA90 became temperature-dependent. There were sudden drops in G′ and G″ for LASA50, LASA75, and LASA90. More specifically, for LASA50 and LASA75, the sudden transition started at around 160 °C, where the G′ dropped from 5.1 × 10^3^ Pa (LASA50, 160 °C) and 1.4 × 10^3^ Pa (LASA75, 160 °C) to 250 Pa (LASA75, 190 °C) and 11 Pa (LASA75, 190 °C). For LASA90, the transition occurred at a lower temperature (around 120 °C), where the G′ decreased from 5.3 × 10^3^ Pa (120 °C) to 10 Pa (190 °C). This can be attributed to the contribution of H-bonding to the entire crosslink density of the LASAs. With a higher COOH concentration, there is a higher percentage of temperature-dependent bonding within the network, therefore lowering the transition temperature. Furthermore, with the increase of the H-bonding concentration in LASAs, intersections were observed in G′ and G″ for LASA75 and LASA90 (defined as gelling temperature), indicating the liquidation of the LASA at an elevated temperature. The gelling temperature for LASA75 was 171 °C and 170 °C for LASA90. These results were consistent with the trends observed for the tan *δ* and viscosities of the LASAs.

**Figure 11:**
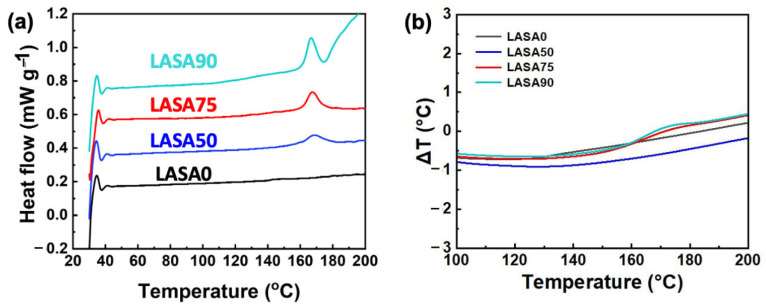
The DSC curves of LASA as a function of temperature. (**a**) DSC and (**b**) DTA.

The rheology results suggested that the re-ROP was prohibited in LASA0, and the residual COOH was a prerequisite for re-ROP. To further confirm this finding, the thermal properties of the LASA networks were evaluated by DSC and DTA (Figure 11). Correspondingly, no heat flow or temperature difference was observed for LASA0 at 160–180 °C in the DSC and DTA curves, suggesting that no chemical reaction occurred in this temperature range. Meanwhile, with the increase of residual COOH in formulation, the peaks for LASA50, LASA75, and LASA90 were rather apparent within the range of 160–180 °C in the DSC and DTA results. As revealed by previously reported literature, the re-ROP of the disulfide ring mostly likely occurred at the 160–180 °C range [28]. Herein, the LASA50, LASA75, and LASA90 underwent re-ROP in the range of 160–180 °C while the re-ROP was prohibited in LASA0.

**Figure 12:**
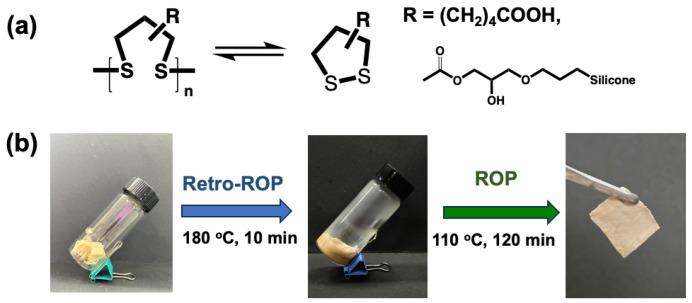
(**a**) The reversibility of (**b**) Photos of the recycling of LASA90 polymers under the heat condition.

Recycling the LASA90 was achieved by ROP and re-ROP of the five-membered disulfide ring. As shown in Figure 12, LASA90 liquefied when heated to a high temperature (180 °C, 10 min) due to the re-ROP. Furthermore, the recycled pastes can be processed into an intact material when ROP happens. Therefore, on-demand recycling of the LASA90 was easily achieved and the recycled pastes can be polymerized into new materials for new applications.

The designed LASA presents rapid reusability, and the incorporation of natural components promotes the development of synthetic silicone materials for use in a more sustainable future. Compared to the reported adhesives synthesized with an *ɑ*-lipoic acid natural linker, the shear strength of LASA90 was higher than those reported in the literature (Table S2) [29,39]. The reported examples of the novel recyclable silicone adhesives demonstrated similar shear strength as LASA90 [40,41,42]. However, in spite of the above-mentioned benefits of the designed LASAs, the shear strengths of the LASAs were lower than the commercialized silicone adhesives and the conventional additional cure silicone adhesives. This is mainly ascribed to the fillers within the formulation that boosted the mechanical properties [1,43,44,45]. We hypothesized that developing LASA compactable fillers that present affinities to both silicone and the *ɑ*-lipoic acid polymer should be the focus of future work.

#### 3.4.2. Thermal Stability of the LASAs

**Figure 13:**
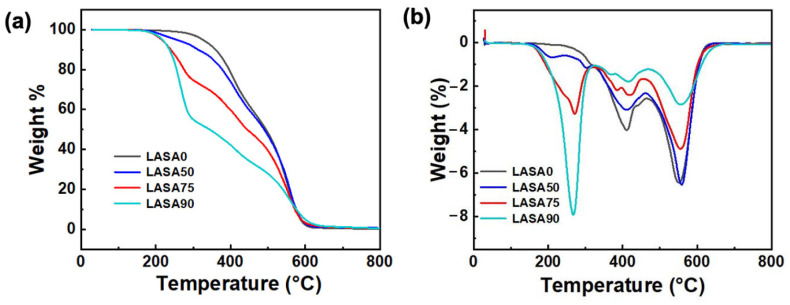
Thermal behaviors of the LASAs from R.T. to 800 °C. (**a**) TGA, (**b**) DTG.

The influence of COOH on the recycling capability of LASAs is rather apparent. To further review the influence of residual COOH on thermal stability under temperatures higher than 200 °C, the TGA and DTG curves of the LASAs were investigated. According to Figure 13, the 5% weight-loss temperature (T_d5%_) was 325 °C, and the major thermal degradation stages were 410 °C and 555 °C, according to the DTG curve. As previously reported, the first degradation peak at 410 °C in DTA was degradation through a series of reactions such as “back-biting” [46]. The degradation peak at 550 °C originated from the cleavage of Si–CH_3_ [47]. Herein, the thermal stability of LASA0 displayed a similar thermal behavior to that of conventional silicone materials. The 5% weight-loss temperatures (T_d5%_) of LASA50, LASA75, and LASA90 were 255 °C, 215 °C, and 217 °C, respectively. The first degradation peak in the DTG figure showed at 267 °C, close to the reported degradation for polylipoic acid. The thermal degradation of the LASAs with residual COOH occurred at a lower temperature than the LASA0. These observations are consistent with the previously reported literature, where the substituent at the COOH sites could inhibit the thermal degradation of the lipoic acid polymer derivatives.

## 4. Conclusions

In summary, we utilized *α*-lipoic acid linked with silicone in the chains as silicone adhesives. This strategy not only induces dynamic bonding with the silicone network, giving the resultant LASA reversible adhesion, thermoplastic properties, and cycling capability, but also reduces the environmental impact of the synthetic polymer by incorporating natural products. The COOH provided universal adhesion between the metallic (steel and aluminum), hydrophilic substrates (glass), and the fluorinated groups in PTFE via metal coordination, H-bonding, and Van der Waals force. Due to the existence of disulfide linkages and the H-bonding between the COOH, the crosslinked network presented dynamic properties, granting the silicone adhesive reversible adhesion and thermoplastic properties in mild conditions. The rheology, DSC, and DTA results confirmed the occurrence of re-ROP at 160–180 °C, and the resulting adhesive displayed rapid on-demand degradation for recycling after use. As a proof of concept, this work not only provided a greener silicone by using a natural product, *α*-lipoic acid, as a component, but also created a silicone adhesive with multiple usages and recycling capability. The adhesion strength is relatively weak when compared to traditional silicone adhesives. We envision developing compatible fillers as a significant aspect of future work. Herein, we expect that the designed silicone adhesive would open up a new avenue for next-generation silicone materials toward a sustainable future.

## Data Availability

Data are contained within the article.

## Supplementary Materials

The following supporting information can be downloaded at: https://www.mdpi.com/article/10.3390/polym16233254/s1, Figure S1 ^1^HNMR of mono-PDMS-H. Figure S2 ^1^H NMR of mono-PDMS-E. Figure S3 ^1^H NMR of bis-PDMS-H. Figure S4 ^1^H NMR of bis-PDMS-E. Figure S5 Schematic diagram of the synthesis of LASA networks. Figure S6 Photo of the LASA90-3 repaired with mono-PDMS-E/bis-PDMS-E = 80:20 in the formulation. Figure S7 LASA0 pre-cure reaction mixture heated at different conditions. Table S1: Summary of formulations for LASA. The calculation method that determines the ratio of mono-PDMS-E/bis-PDMS-E. The calculation method of the residual COOH% in LASAs. Table S2: Adhesive performance of the adhesives synthesized in this work compared to some representative examples reported in previous literature.
