# Supplementary material for: Exploring α-Lipoic Acid Based Thermoplastic Silicone Adhesive: Towards Sustainable and Green Recycling
Source: Polymers (Basel). 2024 Nov 22;16(23):3254. doi: 10.3390/polym16233254 (PMC11644813; doi:10.3390/polym16233254)
Supplement: Supplementary file 1 [file polymers-16-03254-s001.zip › polymers-3269767-supplementary.pdf]

Supplementary Materials

Exploring α-Lipoic Acid Based Thermoplastic Silicone Adhesive: Towards Sustainable and Green Recycling

Jiaqi Wang, Zhaoyutian Chu, and Sijia Zheng *

Key Laboratory of Advanced Textile Materials and Manufacturing Technology and Engineering Research Center for Eco-Dyeing &Finishing of Textiles, Ministry of Education, Zhejiang Provincial Engineering Research Center for Green and Low-carbon Dyeing & Finishing Zhejiang Sci-Tech University, Hangzhou 310018, China

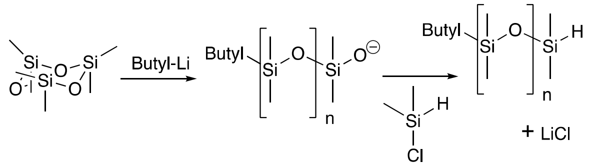

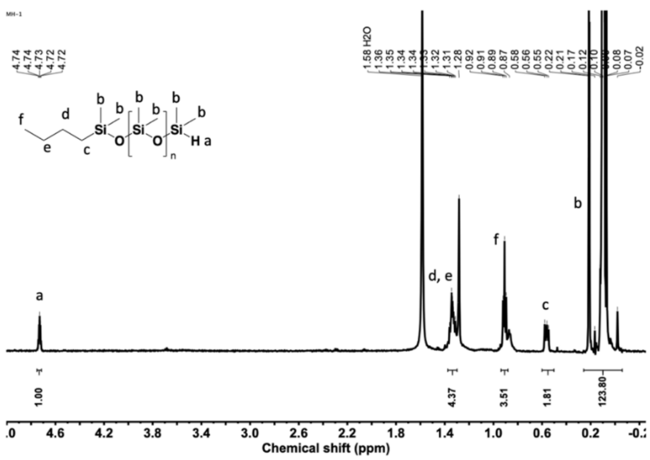

Figure S1. ^{1}HNMR of mono-PDMS-H. The deuterium solvent was $CDCl_3$ and the impurity peak at 1.56 ppm was associated to the H_2O within the deuterium solvent.

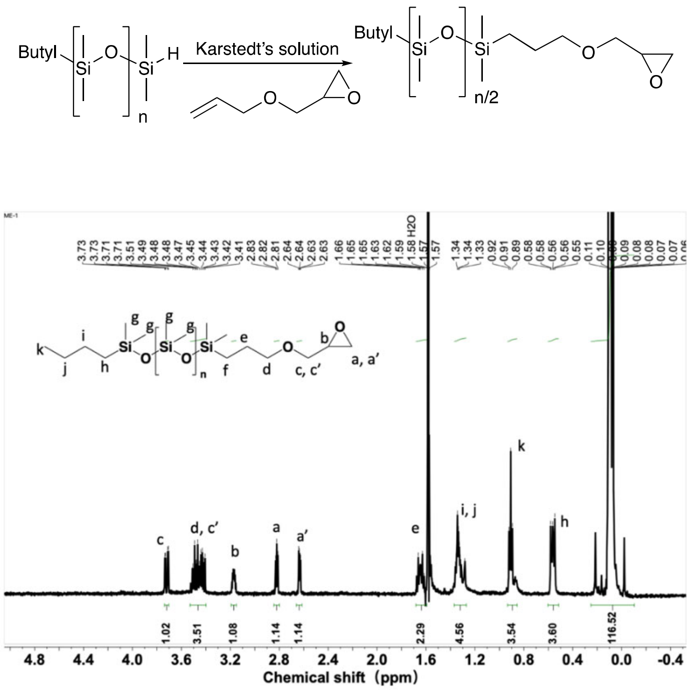

Figure S2. ^{1}H NMR of mono-PDMS-E. The deuterium solvent was $CDCl_3$ and the impurity peak at 1.56 ppm was associated to the H_2O within the deuterium solvent.

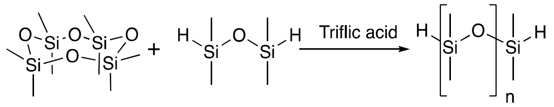

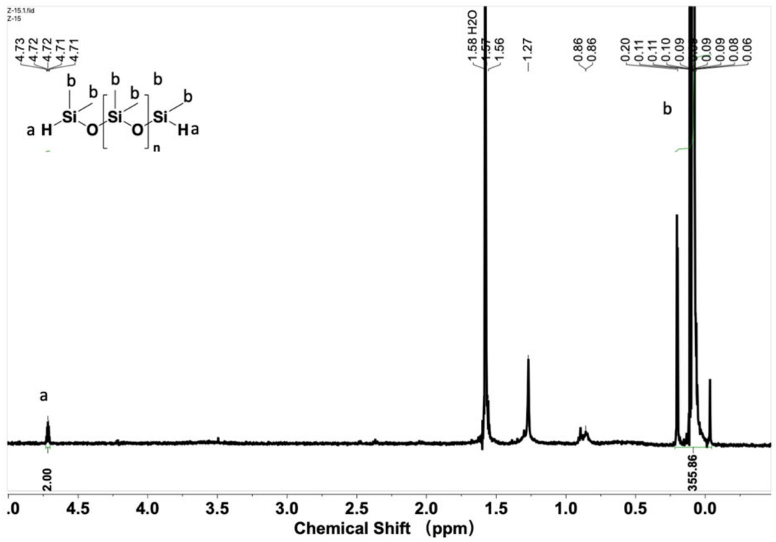

Figure S3. ^{1}H NMR of bis-PDMS-H. The deuterium solvent was $CDCl_3$ and the impurity peak at 1.56 ppm was associated to the H_2O within the deuterium solvent.

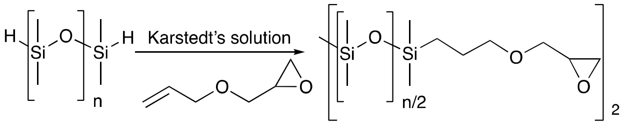

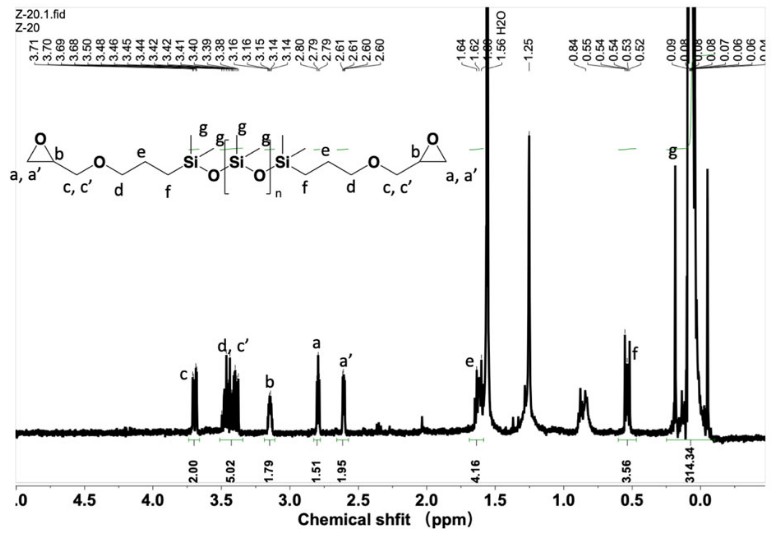

Figure S4. ^{1}H NMR of bis-PDMS-E. The deuterium solvent was $CDCl_3$ and the impurity peak at 1.56 ppm was associated to the H_2O within the deuterium solvent.

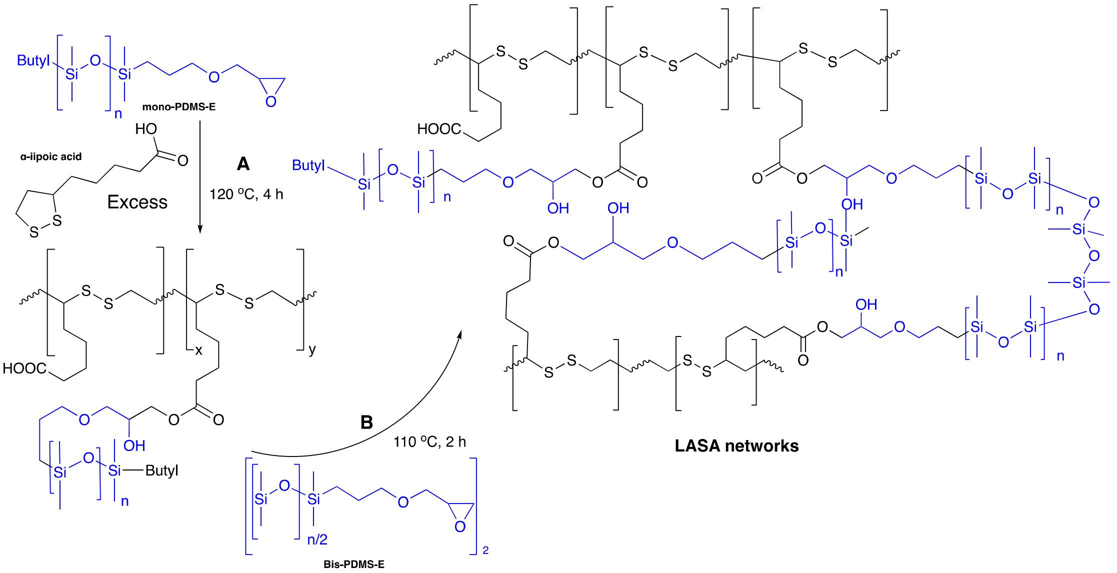

Figure S5. The schematic diagram of the synthetic steps for LASAs

Table S1. Summary of formulations for LASA.

Entry	Samples LASAs	Parameters				Results	
		lipoic acid (g)	mono-PDMS-E (g)	bis-PDMS-E (g)	epoxide ratio from mono-PDMS-E/bis-PDMS-E	residual COOH in network versus total COOH (%)	Shear shear strength [a] (kPa)
1	LASA0	0.1949	1.00	1.55	50/50	0	NA [b]
2	LASA50	0.4874	1.00	1.55	50/50	50	46 ± 5
3	LASA75	0.9748	1.00	1.55	50/50	75	59 ± 3
4	LASA90	2.4370	1.00	1.55	50/50	90	84 ± 5
5	LASA90-2	2.4370	0.46	2.13	20/80	90	45 ± 11
6	LASA90-3	2.4370	1.83	0.53	80/20	90	NA [c]

Note： [a] The shear tests were measured with steel substrates. [b] The LASA0 did not adhere to steel substrates. Therefore, the shear test was not applicable. [c] The LASA90-3 was a liquid. Therefore, the shear test was not applicable.

The epoxide ratio from mono-PDMS-E/bis-PDMS-E was calculated with the following equation:

$$Mols\ of\ expoxie\ from\ mono-PDSM-E = \frac{\frac{m_{mono-PDMS-E}}{Mw_{mono-PDMS-E}}}{\frac{m_{mono-PDMS-E}}{Mw_{mono-PDMS-E}} + \frac{m_{bis-PDMS-E}}{Mw_{bis-PDMS-E}} \times 2} \times 100\%$$

where $m_{mono-PDMS-E}$, and $m_{bis-PDMS-E}$ are the mass of mono-PDMS-E, bis-PDMS-E, respectively. $Mw_{mono-PDMS-E}$ and $Mw_{bis-PDMS-E}$ are the moles of the mono-PDMS-E, bis-PDMS-E, and lipoic acid, respectively.

The residual COOH percentage in the network/ total COOH in formulation was calculated with the following equation:

$$Mols\ of\ expoxie = \frac{m_{mono-PDMS-E}}{Mw_{mono-PDMS-E}} + \frac{m_{bis-PDMS-E}}{Mw_{bis-PDMS-E}} \times 2$$

$$Mols\ of\ COOH = \frac{m_{lipoic\ acid}}{Mw_{lipoic\ acid}}$$

$$Residual\ COOH\ \% \ = \frac{\left(\frac{m_{lipoic\ acid}}{Mw_{lipoic\ acid}} - \frac{m_{mono-PDMS-E}}{Mw_{mono-PDMS-E}} - \frac{m_{bis-PDMS-E}}{Mw_{bis-PDMS-E}} \times 2\right)}{\frac{m_{lipoic\ acid}}{Mw_{lipoic\ acid}}} \times 100\%$$

where $m_{mono-PDMS-E}$, $m_{bis-PDMS-E}$, and $m_{lipoic\ acid}$ are the mass of mono-PDMS-E, bis-PDMS-E, respectively. $Mw_{mono-PDMS-E}$, $Mw_{bis-PDMS-E}$, and $Mw_{lipoic\ acid}$ are the moles of the mono-PDMS-E, bis-PDMS-E, and lipoic acid, respectively.

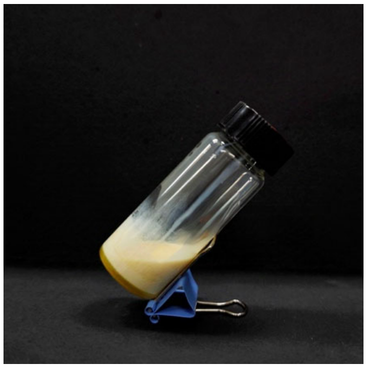

Figure S6. Photo of the LASA90-3 repaired with mono-PDMS-E/ bis-PDMS-E = 80:20 in the formulation. The reaction mixture remained a liquid after heating. This could be explained by the insufficient crosslinking due to the relatively lower difunctional PDMS (bis-PDMS-E) in the formulation.

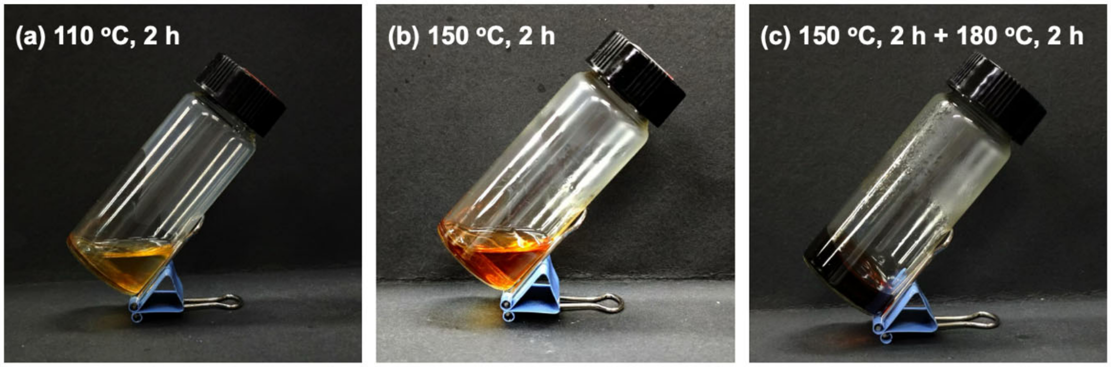

Figure S7. LASA0 pre-cure reaction mixture heated at different conditions.

Table S2. Adhesive performance of the adhesives synthesized in this work compared to some representative examples reported in previous literature.

Adhesive type	Recycle capability or self-healing performance	Dynamic bonding	Filler	Shear strength	Reference
LASA90	Yes	H-bonding, disulfide	No	88 kPa	This work
poly(TA-DIB-Fe)	Yes	disulfide, H-bonding, metal coordination	No	81 kPa	29
Metal lipoate adhesives	Not examined	disulfide, H-bonding, metal coordination	No	10 kPa	39
LM/polyborosiloxane elastomer	Yes	boron-oxygen (B-O) dative bond,	Liquid metal	122 kPa	40
Siliane/ethylene/vinyl acetate (EVA) composite hot melt adhesive	Yes	H-bonding, metal	No	726 kPa	41
Catechol based green silicone adhesive	Yes	imine bonds	No	720 kPa	42
UV curable silicone adhesive	No	-	MQ resin	2 MPa	1
DOWSIL™ Q2-7566 and DOWSIL™ 282	No	No	Kaolin-BASF	320 kPa	43
Addition curing silicone adhesives	No	No	Fumed silica	6.8 MPa	44
Graphene/silicone composite adhesive	No	No	Graphene and nano SiO_2	1.78 MPa	45
